# Effects of Exercise-Based Telerehabilitation Programs on Functional Recovery and Related Outcomes After Stroke: A Systematic Review

**DOI:** 10.3390/healthcare14060741

**Published:** 2026-03-14

**Authors:** Yaiza Casas-Rodríguez, Carlos López-de-Celis, Sergi Rodríguez-Rodríguez, Maria Nicolás-Sola, Gala Inglés-Martínez, Anna Escribà-Salvans

**Affiliations:** 1 Research Group on Methodology, Methods, Models and Results of the Social and Health Sciences (M3O), Faculty of Health and Welfare Sciences, University of Vic-Central University of Catalonia (UVic-UCC), 08500 Vic, Spain; yaiza.casas@uvic.cat (Y.C.-R.); anna.escriba1@uvic.cat (A.E.-S.); 2 Study Group on Pathology of the Locomotor System in Primary Care (GEPALAP), Jordi Gol i Gurina University Institute for Primary Health Care Research Foundation (IDIAPJGol), 08007 Barcelona, Spain; 3 Primary Health Care, Institut Català de la Salut, 08007 Barcelona, Spain; mnicolas.apms.ics@gencat.cat (M.N.-S.); gingles.apms.ics@gencat.cat (G.I.-M.); 4 Department of Physiotherapy, Faculty of Medicine and Health Sciences, Universitat Internacional de Catalunya, 08195 Sant Cugat del Vallès, Spain; 5 ACTIUM Functional Anatomy Group, Faculty of Medicine and Health Sciences, Universitat Internacional de Catalunya, 08195 Sant Cugat del Vallès, Spain; 6 Department of Medicine, Faculty of Medicine and Health Sciences, Universitat Internacional de Catalunya, 08195 Sant Cugat del Vallès, Spain; 7 Center for Research in Health and Social Assistance (CESS), University of Vic-Central University of Catalonia (UVic-UCC), 08500 Vic, Spain; 8 Institute for Research and Innovation in Life and Health Sciences of Central Catalonia (IRIS-CC), University of Vic-Central University of Catalonia (UVic-UCC), 08500 Vic, Spain

**Keywords:** stroke, telerehabilitation, recovery of function, physical therapy modalities, quality of life, activities of daily living, therapeutic exercise

## Abstract

**Background/Objectives**: Stroke is a leading cause of long-term disability, resulting in motor and functional impairments that compromise independence and quality of life. Telerehabilitation offers a promising solution by providing remote, continuous, and accessible post-stroke therapy. This systematic review examined the effects of telerehabilitation on functional capacity, mobility, balance, and quality of life in stroke survivors. **Methods**: A systematic search was conducted following PRISMA guidelines and registered in PROSPERO (CRD420251169784). Searches in PubMed, Cochrane Library, PEDro, Web of Science, Scopus and CINAHL ultimately identified randomized controlled and quasi-experimental trials from the last decade involving adult stroke patients receiving exercise-based telerehabilitation. Methodological quality was assessed using Joanna Briggs Institute tools and Cochrane risk of bias evaluation. Twenty-one studies with a total of 1067 participants were included, featuring supervised tele-sessions, autonomous exercises, caregiver-assisted training, and hybrid approaches. **Results**: Results demonstrated significant improvements in functional capacity, motor performance, balance, and quality of life, comparable to conventional rehabilitation. Additional benefits included enhanced self-efficacy, treatment adherence, and caregiver satisfaction. Overall risk of bias was low, though participant blinding was unfeasible. **Conclusions**: Telerehabilitation may represent a strategy for post-stroke recovery, with studies suggesting outcomes comparable to conventional face-to-face rehabilitation while enhancing accessibility and psychosocial well-being. However, further well-designed, standardized trials with longer follow-up periods are required to confirm its clinical effectiveness.

## 1. Introduction

Stroke is one of the leading causes of acquired disability in adults [1], with a significant impact on both functional autonomy and quality of life [2]. Despite advances in acute treatment, a high proportion of stroke survivors experience persistent sequelae, particularly motor deficits in the upper and lower limbs, balance impairments, and limitations in mobility and activities of daily living [3]. These sequelae create a continuous need for rehabilitative intervention that must not only be intensive and sustained over time, but also adaptable to the different phases of recovery and accessible to all patients, regardless of their geographic or social context [4,5].

Although in-person rehabilitation has become an essential component of post-stroke recovery, its effective implementation faces numerous limitations [6]. The availability of specialized services, waiting times, transportation costs, and the lack of continuity following hospital discharge hinder many patients from receiving sufficiently intense and sustained interventions. This is particularly critical during the transition to home from hospital to home, where the intensity of treatment often decreases [7].

In this context, telerehabilitation has emerged as an innovative alternative for delivering remote interventions through digital technologies [8,9,10,11]. Through interactive platforms, remotely supervised sessions, or self-guided programs, telerehabilitation enables access to therapy beyond the clinical setting, promotes continuity of care and, in many cases, improves patient adherence to treatment [12,13,14]. Moreover, current reference frameworks in motor rehabilitation emphasize the need for intensive, repetitive, task-oriented interventions that are tailored to each phase of the recovery process—principles that can be effectively implemented through well-designed telerehabilitation in the home setting [11,12,13].

Despite the growing interest and expansion of these interventions, the available evidence remains heterogeneous [14]. There are significant differences among studies in terms of the technologies employed, the types of exercises proposed, the dosage of programs, and the outcome measures employed. This variability hampers both the comparative interpretation of results and their systematic application in clinical practice [8,15,16].

This heterogeneity is also reflected in prior evidence syntheses. Although previous systematic reviews have examined telerehabilitation after stroke [8,9,16,17], most prior reviews have synthesized digital or technology-assisted interventions broadly, without restricting inclusion to structured therapeutic exercise protocols targeting activities of daily living-related functional capacity [8,9,16]. Many have included heterogeneous intervention types (e.g., cognitive, speech, or multidisciplinary programs) without specifically focusing on structured therapeutic exercise aimed at motor and functional recovery [8,9,12].

Furthermore, few reviews have clearly distinguished primary functional capacity outcomes from related secondary domains or applied a hierarchical synthesis framework. Consequently, there remains a need for a focused synthesis that specifically examines exercise-based telerehabilitation interventions and prioritizes clinically meaningful functional outcomes. The present review seeks to address these gaps by exclusively analyzing exercise-based telerehabilitation interventions, prioritizing activities of daily living-related functional recovery as the primary outcome and providing a structured narrative synthesis with an assessment of certainty of evidence.

Accordingly, this review adopts a hierarchical analytical framework. The primary objective of this systematic review is to comprehensively examine the characteristics of exercise-based telerehabilitation interventions implemented for post-stroke recovery. Building on this central focus, the review further analyzes key variables related to the intervention, such as exercise type, structure, dosage, progression, and supervision, in addition to the assessment instruments used and the outcomes reported. Particular emphasis is placed on functional recovery as the primary outcome, together with related domains including balance, mobility, quality of life, and psychosocial variables.

## 2. Materials and Methods

### 2.1. Registration

The systematic review was performed in accordance with the Preferred Reporting Items for Systematic Reviews and Meta-Analyses (PRISMA) statement checklist [18]. The systematic review protocol was registered in the PROSPERO database with ID: CRD420251169784 (24 November 2025).

### 2.2. Information Sources and Search

The search strategy was developed following the PICO [19] (Population, Intervention, Comparison, Outcomes) framework.

- Population: Adults who have experienced stroke (ischemic or hemorrhagic) at any stage of recovery (acute, subacute or chronic).
- Intervention: Exercise-based telerehabilitation programs aimed at improving motor function, mobility, strength, balance, functional independence, or quality of life. The review focused specifically on structured therapeutic exercise interventions targeting motor and functional recovery. Studies were not restricted based on the professional discipline delivering the intervention; rather, eligibility was determined by the presence of a core exercise component oriented toward motor and functional outcomes.
- Comparison: Conventional in-person rehabilitation, no additional intervention, or other non-technological forms of care (as defined by the included studies).
- Outcomes: Improvement in physical function (motor skills, gait, upper-limb function), functional capacity (ADLs), health-related quality of life, adherence to treatment, balance, and independence.

The search strategy combined controlled vocabulary (MeSH terms) and free-text keywords to maximize sensitivity.

The search covered studies published between 1 January 2015 and 31 December 2025 and was conducted in January 2026.

Keywords used to develop the search strategy are shown in Table 1.

The databases used in this current systematic review were PubMed, Cochrane Library, PEDro, Web of Science, Scopus and CINAHL ultimate. Furthermore, the reference lists of the included studies were screened to identify additional studies meeting the inclusion criteria. The final search was conducted in January 2026. A complete PubMed database search strategy example is shown in Table 2.

### 2.3. Eligibility Criteria and Study Selection

The inclusion criteria for studies included in this systematic review were as follows: (1) studies published between 1 January 2015 and 31 December 2025, (2) written in Spanish or English, and (3) involving participants aged 18 years or older were included, with (4) no restrictions regarding sex or geographic location. (5) Eligible interventions consisted of structured therapeutic exercise delivered through telerehabilitation specifically targeting motor and functional outcomes. (6) Comparators could include conventional physiotherapy, usual care, or no intervention. (7) Outcomes had to report improvements in functional capacity related to activities of daily living (ADLs), assessed using validated scales such as the Barthel Index or the Functional Independence Measure (FIM). (8) Eligible study designs included randomized controlled trials (including pilot and feasibility RCTs) and controlled or non-randomized intervention studies with pre–post or parallel-group designs.

For the purpose of this review, quasi-experimental studies were defined as intervention studies lacking full randomization but including a comparator group or a pre–post evaluation of outcomes.

Studies were excluded if they did not involve exercise-based telerehabilitation, did not include adult stroke populations, did not report outcomes related to functional capacity in ADLs, or lacked a controlled or quasi-experimental design. Protocols, systematic reviews, case reports, and conference abstracts were also excluded.

Titles and abstracts were screened by two independent authors (Y.C.-R. and C.L.-d-C). In case of a discrepancy, a third author (SRR) was consulted. The Cohen’s Kappa index was used to assess the inter-rater agreement. Landis et al. [20] categorized the Kappa statistic as <0.00 as poor inter-rater agreement, 0.00–0.20 as slight, 0.21–0.40 as fair, 0.41–0.60 as moderate, 0.61–0.80 as substantial, and 0.81–1.00 as almost perfect inter-rater agreement.

### 2.4. Data Collection Process

Data were extracted independently by two reviewers (Y.C.-R. and C.L.-d-C.) using a standardized data extraction form developed for this review. Discrepancies were resolved through discussion and, when necessary, consultation with a third reviewer (S.R.R.).

The following data were extracted for studies included in this systematic review: (a) author’s last name and year of publication; (b) sample size; (c) country where the study was conducted; (d) characteristics of participants; (e) type of stroke; (f) time since stroke onset; (g) type of telerehabilitation intervention; (h) type of exercises performed; (i) dosage and duration of the intervention; (j) main outcomes assessed; (k) measurement instruments used; and (l) main results reported.

### 2.5. Outcomes

The primary outcome selected for this review was functional capacity, as assessed by validated instruments, with particular emphasis on the Barthel Index [21], and the FIM [22], due to their widespread use and clinical relevance in stroke rehabilitation. Functional capacity refers to a patient’s ability to (ADLs) independently or with assistance.

Related (secondary) outcomes included additional aspects of motor and functional recovery, such as upper and lower-limb motor performance, balance, gait, quality of life, treatment adherence, and psychosocial variables. Berg Balance Scale (BBS) [23], Timed Up and Go (TUG) [24], and others, depending on the individual study.

For each included study, outcomes were extracted and categorized according to their relevance to functional recovery, with particular attention given to changes in ADL performance post-intervention.

### 2.6. Data Synthesis

Due to substantial heterogeneity in intervention protocols, outcome measures, and study designs, a quantitative meta-analysis was not performed. Instead, a structured narrative synthesis was conducted.

Results were grouped according to the primary outcome (functional capacity in ADLs) and related outcome domains (motor performance, balance, gait, quality of life, psychosocial variables, and treatment adherence).

The direction of effect and statistical significance reported in each study were analyzed to identify consistent patterns across studies. Conclusions were derived from the collective interpretation of individual study findings within each outcome domain.

### 2.7. Risk of Bias of Individual Studies

The risk of bias of the included studies was assessed by evaluating key methodological domains relevant to telerehabilitation interventions. These domains included random sequence generation, allocation concealment, blinding of participants and personnel, blinding of outcome assessment, incomplete outcome data, and selective reporting.

Each domain was classified as presenting a low, high, or unclear risk of bias based on the methodological information reported in the individual studies. This assessment was conducted to support the interpretation of the findings and to inform the evaluation of the certainty of the evidence in the subsequent synthesis.

### 2.8. Certainty of Evidence (GRADE)

The certainty of the evidence was assessed using the GRADE (Grading of Recommendations Assessment, Development and Evaluation) approach. This assessment was applied to outcomes considered critical for clinical decision-making, including motor function, balance, activities of daily living, and quality of life.

In accordance with GRADE guidelines [25], the certainty of evidence for each outcome was rated as high, moderate, low, or very low, taking into account domains such as risk of bias, inconsistency, indirectness, imprecision, and publication bias. The results of the GRADE assessment are summarized in the corresponding evidence profile table.

## 3. Results

### 3.1. Study Selection

The literature search identified a total of 208 records (PubMed: 11; PEDro: 13; Cochrane Library: 30; Scopus: 73; CINAHL Ultimate: 63; Web of Science: 18). After removing 18 duplicates, 190 records remained for screening. Following title and abstract screening, 169 records were excluded for the following reasons: 27 were systematic reviews, 39 did not involve telerehabilitation interventions, 42 did not include a stroke population, 11 were randomized controlled trial protocols, and 50 were excluded due to not meeting predefined eligibility criteria, including absence of functional capacity outcomes related to ADLs, non-exercise-based interventions, ineligible study designs, or insufficient methodological reporting. Consequently, 21 full-text articles met the inclusion criteria and were included in the qualitative synthesis.

**Figure 1:**
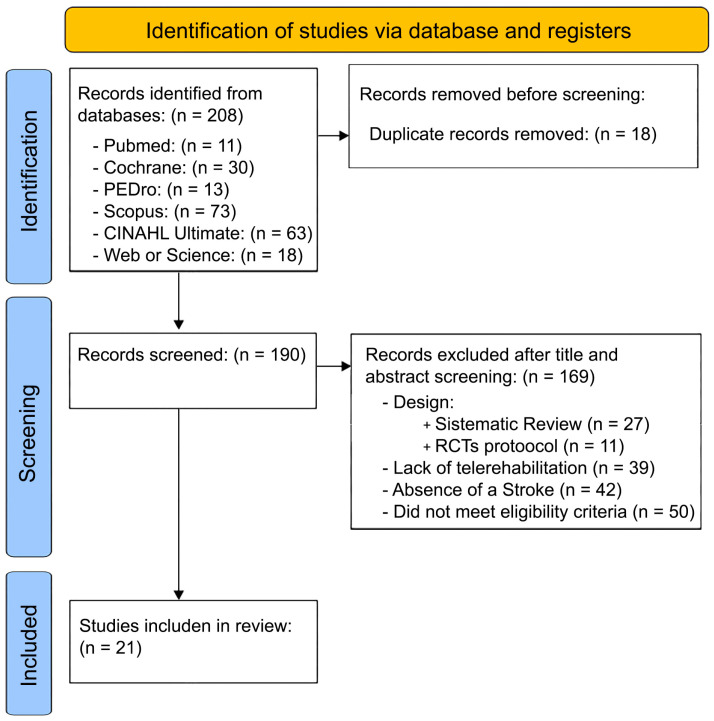
Preferred Reporting Items for Systematic Reviews and Meta Analyses (PRISMA) flow diagram.

The detailed study selection and reasons for excluded articles can be found in the PRISMA flow chart (Figure 1).

### 3.2. Study Characteristics

The included studies comprised a heterogeneous sample of adult stroke survivors. Overall, the studies involved a total of 1067 participants, with ages ranging from 18 to 99 years, and covered different stages of recovery, from the early post-acute period to the chronic phase. Most studies included patients with ischemic and/or hemorrhagic stroke [26,27,28,29,30,31,32,33,34,35,36,37,38,39,40,41,42,43,44,45,46], although some focused on specific subgroups, such as individuals with first-ever subcortical stroke [33] or patients with hemiplegia [30,33,37], while in other cases the stroke subtype was not explicitly reported [34,35,39]. Regarding the post-stroke stage, interventions were implemented during the acute [37,40,41], subacute [30,31,32,33,38], and chronic phases [26,34,35,36,38,40,42,43], with a predominance of studies conducted in the subacute and chronic stages, reflecting the growing clinical interest in extending rehabilitation beyond hospital discharge.

The content of the interventions was diverse and targeted different motor domains. The most frequently evaluated programs combined task-oriented motor training, functional exercises for the upper and lower limbs, balance and gait training, and practice of activities of daily living [26,28,29,30,31,32,33,34,35,36,37,38]. Several studies incorporated technological components, such as EMG-triggered neuromuscular electrical stimulation [30,33], wearable devices for gait training and physiological monitoring [32,37], interactive exergaming systems [26,35,36], or digital platforms and applications for exercise prescription and follow-up [27,31,38,39]. In some trials, more advanced approaches were used, including robot-assisted training [28,29] or virtual reality-based exercises [26,35,36,38,43], aiming to enhance motor learning and treatment adherence.

The telerehabilitation delivery models could be grouped into four main modalities. First, physiotherapist-supervised telerehabilitation, in which exercises were guided and monitored remotely through videoconferencing or telemedicine systems [26,29,30,31,33,37]. Second, technology-supported home-based programs with intensive monitoring, integrating wearable sensors, physiological data transmission, and periodic therapist feedback [32,33,37]. A third modality comprised semi-autonomous or autonomous programs, in which patients performed exercises independently using digital platforms, applications, or exergaming systems, with limited direct supervision [34,35,38,39]. Finally, hybrid or comparative models were identified, combining telerehabilitation with conventional face-to-face care or directly comparing different modes of intervention delivery [28,29,30,33,40].

Program duration and intensity varied considerably across studies. Short-term interventions of up to four weeks were characterized by frequent sessions of moderate duration [26,27,34,38,43], whereas intermediate- and longer-term protocols lasting between five and twelve weeks were the most common and combined frequent training sessions with structured remote supervision or telemonitoring systems [29,30,31,32,33,37]. Some studies implemented high-intensity programs with daily or near-daily sessions [28,29,30,33], while others emphasized continuity of care through sustained home-based interventions over time [32,35,39,40].

A wide range of outcome measures was used to capture the multidimensional effects of telerehabilitation. Motor recovery and functional performance were the most frequently assessed outcomes, using validated instruments such as the Fugl–Meyer Assessment [26,28,29,30,33,36,37,43], the Berg Balance Scale [26,30,31,32,33,34,37,38], the Timed Up and Go test [26,30,32,33,37,38,40], gait parameters [31,32,37,38,40], and measures of activities of daily living, including the Barthel Index and Modified Barthel Index [30,31,32,33,37,40,42]. Upper-limb-specific function was commonly evaluated using standardized tests of motor performance and dexterity, such as the Action Research Arm Test, Wolf Motor Function Test, Nine-Hole Peg Test, and Motor Activity Log [27,28,29,33,36,37,42,43].

In addition, several studies included psychosocial outcomes, such as emotional status, anxiety and depression, self-efficacy, balance confidence, and satisfaction with care [28,32,34,35,37,39], as well as feasibility indicators, including adherence, usability, safety, and patient or caregiver acceptability [27,32,34,35,36,38,39,42,43]. Notably, a subset of trials incorporated neurophysiological or neuroimaging outcomes, including functional connectivity and structural brain measures, to explore mechanisms of motor recovery and neuroplasticity [33].

Overall, the final body of evidence represents a methodologically and clinically diverse set of studies, reflecting the broad applicability of exercise-based telerehabilitation across different stroke populations, intervention modalities, and outcome domains. This heterogeneity highlights the versatility and multidimensional nature of telerehabilitation approaches in post-stroke recovery.

### 3.3. Risk of Bias Assessment

Analysis of the included studies reveals heterogeneity in telerehabilitation interventions, ranging from synchronous videoconferencing systems and mobile applications to home robotic devices and virtual reality. The effects observed in the main clinical domains are detailed below.

The overall assessment revealed a heterogeneous risk of bias across most domains, with certain recurring methodological limitations identified. Random sequence generation and allocation concealment presented a mixed risk profile; although several trials demonstrated low risk in these domains, a considerable proportion [27,36,38,43,44] were judged to be at high risk due to the use of quasi-random or non-randomized designs, while others [40,42,45] presented an unclear risk due to a lack of reporting details.

The largest source of potential bias was related to blinding: blinding of participants and personnel (performance bias) was rated as high risk in almost all studies, an inherent limitation of physical rehabilitation interventions where concealment of active treatment is not possible. Conversely, blinding of outcome assessment (detection bias) predominantly showed a low risk of bias, as most studies employed independent assessors blinded to group assignment.

**Figure 2:**
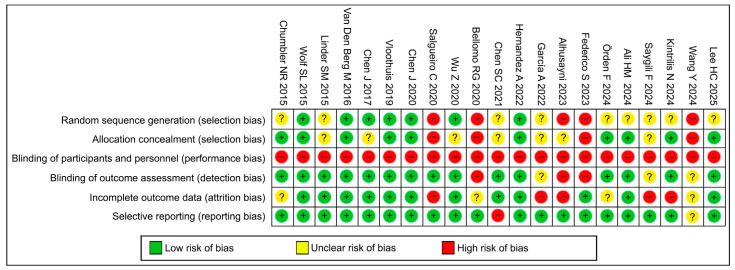
Risk of bias summary [26,27,28,29,30,31,32,33,34,35,36,37,38,39,40,41,42,43,44,45,46].

**Figure 3:**
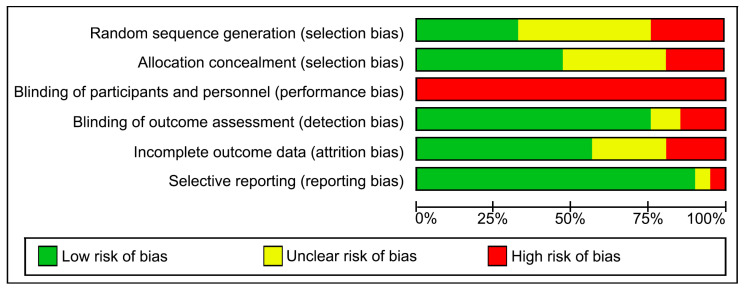
Risk of bias graph.

Regarding attrition bias, most studies provided complete outcome data or used intention-to-treat analyses; however, a small proportion [35,38,40] exhibited a high risk due to significant dropout rates or inadequate per-protocol analyses. Selective reporting was generally considered to pose a low risk in the included studies (Figure 2 and Figure 3).

### 3.4. Certainty of Evidence (GRADE) Assessment

The certainty of the evidence assessed using the GRADE approach was rated as low or very low for all outcomes analyzed (Table 3). For motor function and balance, the certainty of the evidence was judged to be low, mainly due to a serious risk of bias, associated with the consistently high risk of performance bias resulting from the lack of blinding of participants and personnel in most studies [26,27,28,29,30,31,32,33,34,35,36,37,38,39,40,41,42,43,44,45,46] as well as imprecision, related to limited sample sizes and wide confidence intervals. For activities of daily living, the certainty of the evidence was also rated as low, due to a serious risk of bias, particularly relevant for subjective self-reported outcomes, and substantial inconsistency across study results [35,38,40,45]. The certainty of the evidence for quality of life was rated as very low, owing to the combined effect of serious risk of bias, unexplained inconsistency, and imprecision results [35,38,40,44] which substantially limits confidence in the estimated effects. This rating reflects uncertainty in the magnitude and reliability of the effect estimates rather than the absence of reported improvements.

The summary of the main characteristics of the studies is presented in Table 4.

## 4. Discussion

This systematic review provides a comprehensive and updated examination of exercise-based telerehabilitation after stroke, synthesizing evidence from studies that included individuals with ischemic and hemorrhagic stroke across acute, subacute, and chronic phases of recovery, and involving a total of 1067 participants aged 18 to 99 years. Telerehabilitation interventions were delivered through heterogeneous modalities (e.g., therapist-supervised systems, caregiver-mediated models, autonomous digital programs, and hybrid approaches), reflecting the broad applicability of technology-assisted rehabilitation in post-stroke care.

The interpretations presented in this section are derived from the structured narrative synthesis described in Section 2.6, in which outcomes were grouped by domain and interpreted according to the direction and statistical significance of reported effects across studies.

Overall, exercise-based telerehabilitation was reported across the included studies to be associated with improvements in motor function, mobility, balance, activities of daily living, psychosocial well-being, and quality of life. In several cases, outcomes were comparable to conventional rehabilitation when adequate therapeutic intensity, task specificity, and professional supervision were ensured [3,8,13,15,47]. These findings align with contemporary frameworks that emphasize the centrality of structured, goal-oriented, and progressive motor training to maximize recovery [12,14] and with evidence supporting experience-dependent plasticity after neurologic injury [6].

However, given the heterogeneity in intervention protocols and outcome measures, these findings should be interpreted cautiously and within the context of a narrative synthesis rather than pooled quantitative estimates. Results concerning quality-of-life and psychosocial domains should also be interpreted within the context of related (secondary) outcomes, as these measures were not primary inclusion criteria and were variably reported across studies.

The substantial heterogeneity observed across studies warrants careful interpretation of the findings, which is consistent with previous systematic reviews in the field of stroke telerehabilitation [8,9,16]. Variability in intervention protocols (e.g., type of technology used, level of supervision, exercise content, and dosage), stroke phases (acute, subacute, and chronic), and outcome measures limits direct comparability between trials. Such diversity may influence the magnitude and consistency of reported effects, as recovery trajectories and responsiveness to exercise differ across stroke stages and baseline impairment levels [4,15,17].

Due to this clinical and methodological heterogeneity, a quantitative meta-analysis or subgroup analysis was not considered appropriate, as pooling effect estimates under these conditions could produce misleading conclusions. Therefore, a structured narrative synthesis was adopted to allow domain-specific interpretation while preserving methodological transparency.

Clinical responses varied across studies and likely depended on stroke characteristics, baseline impairment, and the content and dose of training. In earlier post-stroke phases, functional gains tended to be more evident in domains such as gait, balance, and ADL performance, which is consistent with the notion of a time-sensitive window of enhanced responsiveness to training and recovery processes [4,12]. In chronic stages, relevant benefits were also observed, particularly when programs promoted high repetitions, functional practice, and engagement—elements that are strongly supported by locomotor and resistance-training recommendations and clinical practice guidelines [3,9,29]. Importantly, the heterogeneity of delivery models suggests that “telerehabilitation” should not be interpreted as a single intervention, but rather as a delivery framework in which outcomes depend on how exercise is prescribed, monitored, progressed, and integrated into daily life [10,13,48,49].

Complementing the clinical synthesis, certainty of evidence was assessed using the GRADE approach. The evidence supporting exercise-based telerehabilitation for stroke survivors was rated as low for motor function, balance, and activities of daily living, and very low for quality of life. Although the majority of the included studies were randomized controlled trials, certainty was downgraded mainly due to a high risk of performance bias related to the lack of blinding of participants and personnel, imprecision associated with small sample sizes and wide confidence intervals, and inconsistency across study results. These findings suggest that exercise-based telerehabilitation may be beneficial for functional recovery and overall well-being; however, larger and more methodologically rigorous trials are required to increase confidence in the magnitude and durability of the observed effects [9,47]. These considerations should be taken into account when interpreting the clinical implications discussed below.

### 4.1. Exercise Characteristics

The studies included in this review applied a wide range of exercise modalities addressing upper-limb function, lower-limb performance, and global mobility, reflecting the diversity of telerehabilitation approaches currently used in post-stroke care. Upper-limb-focused programs primarily involved task-oriented training, repetitive practice of functional movements, fine motor activities, progressive strengthening, and exercises integrated into activities of daily living. These interventions were commonly delivered through digital platforms or interactive telerehabilitation systems [27,28,29,33,35,36,42,45].

Studies such as the HAAPI trial by Wolf et al. and the analyses conducted by Linder et al. showed that high-intensity home-based telerehabilitation programs based on repetitive and functionally meaningful upper-limb practice can significantly improve manual dexterity, spontaneous use of the affected arm, and patient-reported outcomes [28,29]. Similarly, interventions using modified constraint-induced movement therapy delivered via telerehabilitation demonstrated clinically relevant improvements in upper-limb motor function when training was structured, progressive, and remotely supervised [45]. Feasibility studies and pilot trials further indicated that personalized online physiotherapy programs and interactive digital platforms support adherence and facilitate functional recovery in subacute and chronic stroke populations [27,35,36,42].

Lower-limb and whole-body interventions focused mainly on balance exercises, gait training, transfers, lower-extremity strengthening, and coordination or endurance activities [30,31,32,33,37,38,40,41]. Studies such as those by van den Berg et al. and the CARE4STROKE trial by Vloothuis et al. demonstrated that caregiver-mediated exercise programs supported by e-health tools increase practice frequency and lead to improvements in mobility, gait speed, balance, and functional independence. These effects were particularly evident in the acute and subacute phases after stroke [31,32]. Other studies conducted in hospital-based and home-based settings also reported improvements in balance and functional performance when telerehabilitation was delivered within collaborative care models and supported by remote professional supervision [26,37,38,41].

Across all intervention types, outcomes were consistently associated with how the exercise programs were designed and delivered. Programs that included repeated practice, progressive increases in difficulty, and exercises closely related to daily activities achieved better functional results than those with lower structure or intensity [29,30,31,32,33]. In this context, telerehabilitation mainly served to support treatment continuity, organize exercise delivery, and enable remote supervision and feedback, rather than acting as the primary therapeutic element. The heterogeneity of the interventions also highlights the importance of adapting exercise content to the patient’s functional status, stroke phase, and living environment, supporting individualized and activity-centered rehabilitation programs.

### 4.2. Dosage and Intensity

Program duration, training frequency, and exercise intensity varied considerably across the included studies, reflecting the absence of a standardized dosage framework for exercise-based telerehabilitation after stroke. Intervention periods ranged from short programs of three to four weeks to longer protocols lasting up to twelve weeks, with substantial variability in daily training time and weekly frequency.

Several studies implemented high-dose interventions characterized by frequent and prolonged training sessions. For example, the HAAPI trial by Wolf et al. and the subsequent analysis by Linder et al. applied a structured home-based program consisting of three hours of daily training, five days per week, over eight weeks, resulting in a total target dose of approximately 120 h [28,29]. Both studies reported significant improvements in upper-limb motor function and quality of life, despite no clear superiority of robot-assisted therapy over conventional home exercise when total training time was equivalent. These findings suggest that total practice volume and task engagement may be more relevant than the specific delivery modality.

Other studies adopted moderately intensive protocols. Caregiver-mediated interventions such as those by van den Berg et al. and the CARE4STROKE trial by Vloothuis et al. prescribed practice sessions of at least 30 min per day on five or more days per week over eight weeks, yielding an accumulated training dose of approximately 20 to 25 h [31,32]. Although between-group differences were modest for some primary outcomes, these interventions successfully increased overall practice time and were associated with improvements in extended activities of daily living, mobility, and psychosocial outcomes, particularly for caregivers.

Shorter-duration programs were also represented. Several feasibility and pilot studies delivered interventions over four to six weeks, with session durations ranging from 20 to 60 min and frequencies between three and six sessions per week [26,27,34,35,38]. While these studies generally reported significant within-group improvements in motor or balance outcomes, between-group differences were often small or non-significant. This pattern suggests that shorter or lower-dose interventions may be sufficient to induce initial functional gains but may be less likely to produce robust between-group effects.

In contrast, some of the longest and most intensive protocols combined high frequency with extended duration. For instance, Chen J. et al. (2017) applied a 12-week program involving twice-daily physical exercise sessions combined with EMG-triggered neuromuscular electrical stimulation, resulting in a very high cumulative dose [30]. Both the telerehabilitation and conventional rehabilitation groups achieved significant functional improvements, with no between-group differences, indicating that when dosage is matched, telerehabilitation can achieve outcomes comparable to face-to-face care. Similarly, Wang et al. (2024) implemented a 12-week remote program with twice-daily sessions and wearable-assisted gait training, reporting superior outcomes in motor function, balance, gait parameters, emotional status, and neurocytokine levels compared with usual care [44].

Across studies, adherence and effective training exposure varied widely, even within similar protocols. Several trials highlighted substantial interindividual differences in actual usage time, particularly in technology-assisted programs, which appeared to influence clinical outcomes [29,35,36]. Studies that incorporated regular therapist contact, caregiver involvement, or structured monitoring systems tended to report higher adherence and more consistent functional gains [30,31,32].

Overall, the available evidence indicates that higher training volume, achieved through increased session frequency, longer program duration, or both, is generally associated with greater functional improvements. However, the wide heterogeneity in dosage parameters and reporting methods limits the identification of clear dose–response thresholds. These findings underscore the need for individualized dosage prescription in telerehabilitation, balancing intensity, feasibility, patient tolerance, and contextual factors to optimize functional recovery after stroke.

### 4.3. Outcomes Across Domains

The outcomes assessed across the included studies covered a broad range of motor, functional, psychosocial, and quality-of-life domains, reflecting the multidimensional impact of exercise-based telerehabilitation after stroke. Motor recovery and functional performance were the most frequently evaluated outcomes and were consistently measured using validated clinical instruments.

Upper-limb motor function was commonly assessed using tools such as the Fugl–Meyer Assessment for the upper extremity, the Action Research Arm Test, and the Wolf Motor Function Test [28,29,30,33,45]. Studies focusing on upper-limb telerehabilitation generally reported improvements in motor control, dexterity, and functional use of the affected arm. In particular, the HAAPI trial by Wolf et al. and the related analyses by Linder et al. demonstrated significant gains in upper-limb performance and patient-reported outcomes following intensive, home-based telerehabilitation programs [28,29]. Similar improvements were observed in studies applying modified constraint-induced movement therapy and interactive digital platforms, especially when training emphasized repetitive and task-oriented practice [42,45].

Lower-limb function, balance, and gait outcomes were frequently evaluated using the Berg Balance Scale, gait speed tests, the Timed Up and Go, and walking endurance measures [26,30,31,32,34,38,41]. Interventions targeting mobility and balance consistently reported improvements in gait performance, postural control, and functional independence. Studies such as those by van den Berg et al. and the CARE4STROKE trial by Vloothuis et al. showed that caregiver-mediated exercise programs supported by e-health tools were effective in improving balance, mobility, and extended activities of daily living, particularly in the acute and subacute phases after stroke [31,32]. Comparable findings were reported in hospital-based and hybrid models integrating telerehabilitation with conventional care [37,38,41].

Functional independence and activities of daily living were assessed using instruments such as the Barthel Index, the Functional Independence Measure, and stroke-specific ADL scales [30,31,32,33,38]. Improvements in ADL performance were more consistently observed in studies combining motor training with functional task practice and higher training frequency. These gains were evident across different stroke phases but appeared more pronounced in interventions emphasizing lower-limb mobility and whole-body function [30,31,32].

Psychosocial outcomes were also addressed in several studies, including measures of quality of life, emotional status, self-efficacy, and balance confidence [28,31,34,39,43]. Quality of life was commonly assessed using instruments such as the Stroke Impact Scale and EQ-5D, with improvements reported in domains related to physical function, participation, and emotional well-being [28,31,43]. Studies incorporating regular therapist contact, caregiver involvement, or real-time feedback tended to report greater improvements in self-efficacy, balance confidence, and satisfaction with care [31,34,39].

Overall, the outcome patterns observed across studies indicate that exercise-based telerehabilitation can generate meaningful improvements across multiple domains of recovery after stroke. Motor and functional gains were the most consistently reported outcomes, while psychosocial benefits appeared particularly sensitive to program structure, supervision, and patient engagement. These findings support the role of telerehabilitation as a comprehensive intervention capable of addressing not only physical impairments but also functional performance and patient-centered outcomes.

### 4.4. Home-Based and Hybrid Models

The included studies implemented a variety of home-based and hybrid telerehabilitation models, combining remote supervision, caregiver involvement, and integration with conventional rehabilitation services. These approaches primarily aimed to ensure continuity of care after hospital discharge while reducing logistical barriers and maintaining therapeutic intensity.

Supervised home-based programs, supported by videoconferencing and telemonitoring, demonstrated significant improvements in motor function and activities of daily living, with outcomes comparable to conventional outpatient rehabilitation [30]. Similarly, hybrid models combining in-person sessions with remote exercise programs, such as those described in the CARE4STROKE studies [31,32], were associated with improvements in mobility, balance, and functional independence, particularly when exercises were caregiver-mediated.

Caregiver involvement facilitated higher training frequency and supported the transfer of practiced tasks into daily life, which appeared especially beneficial for individuals with lower functional autonomy [31,32]. Across delivery formats, ongoing professional supervision, including regular feedback and remote adjustment of exercise prescriptions, was associated with better adherence and more consistent functional gains compared with fully autonomous interventions [37,39,41].

In summary, the available evidence suggests that home-based and hybrid telerehabilitation models provide an effective and flexible framework for post-stroke rehabilitation when structured programs, caregiver support, and continued professional supervision are ensured.

### 4.5. Comparison with Previous Reviews

When comparing these findings with previous reviews [8,16,47,49], both bodies of evidence indicate that telerehabilitation can achieve outcomes equivalent to conventional rehabilitation. However, earlier reviews focused on highly diverse and nonspecific telehealth interventions. The present review provides a more precise perspective because it examines only exercise-based interventions and describes in greater detail the therapeutic components that influence recovery. The findings show that more intensive and longer programs generate greater improvements in mobility, balance, and overall function [17,28,29,45]. Clear differences also emerge between upper-limb and lower-limb exercise interventions. Upper-limb programs [17,27,28,29,45] lead to gains in dexterity, coordination, and functional use. In contrast, lower-limb programs [26,27,30,31,32] improve gait, stability, and independence in daily activities.

In addition to these stage-specific patterns, this review underscores that recovery trajectories differ depending on the phase of stroke. Patients in the acute and subacute stages experience faster gains in gait and functional performance [30,31,32]. Patients in the chronic stage [17,26,28,34,45] tend to benefit more in domains such as self-efficacy, balance confidence, and upper-limb functional use.

Furthermore, the inclusion of recent studies involving hybrid models and exergaming [26,34] adds an additional layer of insight. These approaches increase motivation and support higher training volumes, which may enhance clinical outcomes. Psychosocial results, often overlooked in previous reviews, are also included. These results show improvements in emotional well-being [28,31], self-efficacy [26,31,34], and caregiver burden [30,31].

### 4.6. Methodological Considerations

The interpretation of the findings should consider the methodological heterogeneity of the included studies. Although most adopted randomized or controlled designs, relevant differences were observed in sample size, intervention structure, outcome selection, and follow-up duration, which affect comparability across studies.

Several studies were based on small samples, particularly pilot trials, which may limit statistical power and increase uncertainty in the observed effects [26,27,34,35,36,41,42,45]. In contrast, trials with larger sample sizes, such as those conducted by Wolf et al., Vloothuis et al., and Chen J. et al., provided more consistent estimates of motor and functional outcomes [28,29,31,33,40,43,44].

Outcome selection was diverse and reflected different therapeutic objectives. Motor function and functional independence were systematically assessed using validated scales such as the Fugl-Meyer Assessment [50], the Barthel Index [21], the Berg Balance Scale [23], the 10-Meter Walk Test [51], and the Timed Up and Go [24]. In addition, several studies [28,34,35,39] included psychosocial outcomes, such as quality of life, self-efficacy, and balance confidence, primarily assessed using instruments such as the Stroke Impact Scale [52], the EQ-5D [53], the Falls Efficacy Scale [54], or balance confidence scales [55], particularly in chronic population. This variability indicates that definitions of therapeutic success were not uniform across trials.

Complete blinding was not feasible in most interventions. However, several studies employed blinded outcome assessors or standardized assessment procedures to reduce detection bias [29,30,31,32]. Intervention delivery models ranged from highly supervised programs, with structured therapist follow-up and frequent feedback, as reported in studies by Wolf et al., Chen J. et al., and Vloothuis et al. [29,30,31]., to more autonomous or caregiver-mediated approaches, such as those described by van den Berg et al., Chumbler et al., and Özden et al. [32,34,39]. These differences likely influenced both the actual training dose and participant engagement.

Taken together, the methodological diversity observed reflects the evolving nature of exercise-based telerehabilitation research. While this heterogeneity limits direct comparison across studies, it also provides valuable insight into how different study designs and delivery models may influence rehabilitation outcomes after stroke.

### 4.7. Final Interpretation

Overall, this review offers a more nuanced and complete understanding of exercise-based telerehabilitation. It identifies several factors that shape clinical outcomes, including the type of exercise performed, the intensity and duration of the program, the phase of stroke, and the psychosocial responses associated with training. Considering these elements, it provides a clearer interpretation of which components are most likely to drive meaningful recovery. Collectively, these findings reinforce exercise-based telerehabilitation as a promising and potentially effective, accessible, and scalable component of post-stroke care. When delivered with sufficient intensity, progression, and supervision, it may support clinically meaningful improvements across motor, functional, psychosocial, and quality-of-life domains in acute, subacute, and chronic stroke survivors. However, the magnitude and consistency of these effects remain subject to methodological limitations in the current evidence base. Its capacity to extend continuity of care into the home environment positions telerehabilitation as a potentially important component in the future landscape of neurorehabilitation.

Therefore, telerehabilitation should be understood not as an inferior substitute, but as a complementary therapeutic approach that extends the reach of post-stroke rehabilitation beyond traditional clinical settings.

Future research should focus on larger and methodologically rigorous trials, with standardized reporting of exercise dosage, adherence, and long-term outcomes. Such studies are needed to define optimal intervention parameters and to support the evidence-informed integration of exercise-based telerehabilitation into routine post-stroke care.

### 4.8. Limitations

Several limitations should be considered when interpreting the findings of this review. First, there was substantial heterogeneity in intervention design, exercise dosage, supervision models, and outcome measures. This variability limits direct comparison between studies and makes it difficult to identify optimal training parameters.

Second, several of the included studies were pilot or feasibility trials with small sample sizes, which may reduce statistical power and increase uncertainty regarding the observed effects. Although larger randomized trials provided more robust evidence, the overall body of evidence remains uneven.

Third, blinding of participants and therapists was not feasible in most interventions due to the nature of exercise-based rehabilitation. While some studies employed blinded outcome assessors or standardized assessment procedures to mitigate detection bias, the risk of performance bias cannot be fully excluded, and some degree of detection bias may persist for certain outcomes.

In addition, variability in the definition of telerehabilitation interventions and in the characteristics of control or usual care conditions may have contributed to heterogeneity in the observed effects. Only studies published in English or Spanish were included, which may have introduced language bias and limited the inclusion of potentially relevant studies published in other languages.

This review may also be subject to methodological limitations inherent to systematic reviews. Although multiple databases were searched, publication bias cannot be fully excluded, as unpublished or non-indexed studies may not have been identified. The heterogeneity of interventions, outcome measures, and study designs precluded quantitative synthesis, limiting the ability to generate pooled effect estimates. These factors may influence the generalizability and strength of the conclusions drawn.

Conclusions regarding quality-of-life and psychosocial outcomes should be interpreted cautiously, as these were not primary eligibility criteria and were inconsistently reported across studies. This may limit the strength of inferences in these domains. Finally, reports on adherence and long-term follow-up were inconsistent. This limitation restricts conclusions regarding the sustainability of treatment effects and the long-term impact of telerehabilitation on functional recovery.

## 5. Conclusions

Exercise-based telerehabilitation appears to be a feasible and potentially effective alternative to conventional post-stroke physiotherapy. Improvements were reported in motor function, mobility, balance, and quality of life, along with high levels of patient satisfaction and adherence.

Although no serious adverse events were reported in the included studies, safety was not a predefined outcome of this review and should therefore be interpreted cautiously.

When programs are well structured, delivered at appropriate intensity, and supported by professional supervision or caregiver involvement, telerehabilitation may achieve outcomes comparable to conventional face-to-face rehabilitation across motor, functional, and psychosocial domains. However, given the heterogeneity of interventions and outcome measures, these findings should be interpreted within the context of a structured narrative synthesis rather than pooled quantitative estimates.

The available evidence suggests that the success of telerehabilitation may depend more on the quality of exercise prescription and implementation than on the specific digital technology used. In this context, telerehabilitation appears to provide a viable means to extend continuity of care, improve access to rehabilitation services, and promote active patient engagement during both early and chronic phases after stroke.

## Figures and Tables

**Table 1:** The keywords used for the search strategy.

Stroke	Therapeutic Exercises	Functional Capacity
Cerebrovascular accident	Physical therapy	Activities of daily living (ADL)
Post-stroke	Physiotherapy	Functional independence
Chronic stroke	Telerehabilitation	Functional recovery
	Telehealth	Barthel Index
	Remote rehabilitation	Functional Independence Measure (FIM)
	Home-based rehabilitation	

**Table 2:** Database and the specific search equations.

Database	Specific Search Equation
PubMed	((“Stroke” [MeSH] OR “cerebrovascular accident” OR “chronic stroke” OR “post-stroke”) AND (“Exercise Therapy” [MeSH] OR “therapeutic exercise” OR “physical therapy” OR “physiotherapy”) AND (“Telerehabilitation” [MeSH] OR “tele-rehabilitation” OR “telehealth” OR “remote rehabilitation” OR “home-based rehabilitation”) AND (“Activities of Daily Living” [MeSH] OR “functional capacity” OR “ADL” OR “functional recovery” OR “functional independence” OR “Barthel Index” OR “Functional Independence Measure” OR “FIM”)) AND (“adult” [MeSH] OR “aged” [MeSH]) AND (“clinical trial” [Publication Type] OR “controlled clinical trial” [Publication Type] OR “randomized controlled trial” [Publication Type]) AND (“english” [lang] OR “spanish” [lang])
PEDro	Stroke AND exercise AND telerehabilitation AND daily living Neurology Since 2015
Cochrane	(“stroke” OR “cerebrovascular accident” OR “chronic stroke” OR “post-stroke”) AND (“therapeutic exercise” OR “physical therapy” OR “physiotherapy”) AND (“tele-rehabilitation” OR “telehealth” OR “remote rehabilitation” OR “home-based rehabilitation”) AND (“activities of daily living” OR “ADL” OR “functional capacity” OR “functional independence” OR “functional recovery” OR “Barthel Index” OR “FIM”)
CINAHL ultimate	(MH “Stroke+” OR TX “stroke” OR TX “cerebrovascular accident” OR TX “chronic stroke” OR TX “post-stroke”) AND (MH “Therapeutic Exercise+” OR MH “Physical Therapy+” OR TX “exercise therapy” OR TX “therapeutic exercise” OR TX “physical therapy” OR TX “physiotherapy”)AND(MH “Telerehabilitation” OR MH “Telehealth+” OR TX “tele-rehabilitation” OR TX “telehealth” OR TX “remote rehabilitation” OR TX “home-based rehabilitation”)AND(MH “Activities of Daily Living+” OR TX “functional capacity” OR TX “ADL” OR TX “functional recovery” OR TX “functional independence” OR TX “Barthel Index” OR MH “Functional Independence Measure” OR TX “FIM”) AND (PT “Clinical Trial” OR PT “Randomized Controlled Trial”)
Scopus	(TITLE-ABS-KEY(“stroke*” OR “cerebrovascular accident*” OR “chronic stroke” OR “post-stroke”)) AND (TITLE-ABS-KEY(“exercise therap*” OR “therapeutic exercise*” OR “physical therapy” OR “physiotherapy”)) AND (TITLE-ABS-KEY(“telerehabilitation” OR “tele-rehabilitation” OR “telehealth” OR “remote rehabilitation” OR “home-based rehabilitation”)) AND (TITLE-ABS-KEY(“activities of daily living” OR “functional capacity” OR “ADL” OR “functional recovery” OR “functional independence” OR “Barthel Index” OR “Functional Independence Measure” OR “FIM”)) AND (TITLE-ABS-KEY(“clinical trial*” OR “randomized controlled trial*” OR “controlled clinical trial*”))
Web of Science	(TS = (“stroke” OR “cerebrovascular accident” OR “chronic stroke” OR “post-stroke”)) AND (TS = (“Exercise Therapy” OR “therapeutic exercise” OR “physical therapy” OR “physiotherapy” OR “physiotherap*”)) AND (TS = (“Telerehabilitation” OR “tele-rehabilitation” OR “telehealth” OR “remote rehabilitation” OR “home-based rehabilitation”)) AND (TS = (“Activities of Daily Living” OR “functional capacity” OR “ADL” OR “functional recovery” OR “functional independence” OR “Barthel Index” OR “Functional Independence Measure” OR “FIM”)) AND (TS = (“adult*” OR “aged” OR “elderly”)) AND (TS = (“clinical trial” OR “controlled clinical trial” OR “randomized controlled trial” OR “RCT”))

**Table 3:** Summary of findings (GRADE).

Outcome	No. of Studies	Risk of Bias	Inconsistency	Indirectness	Imprecision	Publication Bias	Certainty of Evidence
Motor Function (FMA-UE/LE, WMFT)	12 RCTs (850)	Not serious	Not serious	Serious 2	Undetected	Motor Function (FMA-UE/LE, WMFT)	LOW ⊕⊕◯◯
Balance (BBS, TUG)	6 RCTs (320)	Serious 1	Not serious	Not serious	Serious 2	Undetected	LOW ⊕⊕◯◯
Activities of Daily Living (ADL) (Barthel Index, FIM)	9 RCTs (900)	Serious 3	Serious 4	Not serious	Not serious	Undetected	LOW ⊕⊕◯◯
Quality of Life (SIS, SS-QOL)	7 RCTs (550)	Serious 3	Serious 4	Not serious	Serious 2	Undetected	VERY LOW ⊕◯◯◯

1, Risk of Bias: Downgraded one level due to high risk of performance bias (lack of blinding of participants and personnel) in most studies. 2, Imprecision: Downgraded one level due to small sample sizes in several pilot studies and/or wide confidence intervals crossing the line of no effect. 3, Risk of Bias (Subjective outcomes): Downgraded one level due to lack of blinding in self-reported outcomes (ADL, QoL), making them susceptible to placebo effects or social desirability bias. 4, Inconsistency: Downgraded one level due to unexplained heterogeneity in results across studies estimates. The level of certainty of the evidence: ⊕⊕◯◯ low certainty; ⊕◯◯◯ very low certainty.

**Table 4:** Summarizes the characteristics of the included studies.

Study	Country	Study Design	Participants (n, Stroke Type, Phase)	Methodology (Intervention, Exercises, Dosage)	Outcomes	Assessment Instruments	Main Results
Chumbler NR et al. (2015) [39]	United States	Multisite RCT, prospective, single-blinded	n = 52; ischemic/hemorrhagic; ≤24 months post-stroke (acute–chronic)	3-month home-based telerehabilitation including functional balance, mobility and fall-prevention exercises (3 home visits + telephone follow-up) vs. usual care.	Falls-related self-efficacy; patient satisfaction with care (hospital and home); qualitative experience with telerehabilitation	Falls Efficacy Scale (FES); Stroke-Specific Patient Satisfaction with Care scale (SSPSC: hospital and home subscales); semi-structured qualitative exit interviews	No significant between-group differences in falls self-efficacy. Higher hospital-care satisfaction in intervention group at 6 months (*p* = 0.029); trend for home-care satisfaction (*p* = 0.077). No safety concerns reported.
Wolf SL et al. (2015) (HAAPI Trial) [29]	USA	Prospective, multisite, single-blinded RCT	n = 99; ischemic/hemorrhagic; <6 months post-stroke (subacute)	8-week dose-matched home program (3 h/day, 5 d/week). Robotic upper-limb training (2 h/day) + HEP (1 h/day) vs. HEP alone with remote therapist monitoring	Upper-limb motor function; adherence/feasibility.	Primary: ARAT. Secondary: WMFT, FMA-UE.	Both groups improved significantly (within-group *p* < 0.001). No superiority of robotic + HEP vs. HEP alone (ARAT interaction *p* = 0.147; FMA *p* = 0.594). Some WMFT measures favored HEP-only (*p* = 0.012–0.001). Similar total therapy time; low dropout (<10%).
Linder SM et al. (2015) [28]	United States	Multisite RCT; assessor-blinded; intention-to-treat	n = 99; ischemic/hemorrhagic; <6 months post-stroke (subacute)	8-week home-based upper-limb training (3 h/day, 5 d/week): customized HEP vs. robot-assisted therapy + HEP, with remote monitoring and weekly progression.	Quality of life and depressive symptoms.	SIS; CES-D.	Significant within-group improvements in most SIS domains and CES-D scores. No significant between-group differences (no time × group interaction). Both interventions similarly effective.
Maayken van den Berg (2016) [32]	Australia	Pragmatic pilot RCT (proof-of-concept)	n = 63; ischemic/hemorrhagic; early phase (24 h–3 months)	8-week caregiver-mediated mobility program (≥30 min/session, ≥5 days/week) with e-health/telerehabilitation support vs. usual inpatient and home rehabilitation.	Mobility; ADL; balance; length of stay; readmissions; caregiver outcomes.	Primary: SIS 3.0 (mobility). Secondary: BI, NEADL, BBS, TUG, mRS, LOS.	No between-group difference in primary mobility outcome (*p* = 0.6). Caregivers showed reduced fatigue and increased self-efficacy (*p* < 0.05). Per-protocol analysis showed improved extended ADL (*p* ≤ 0.03), shorter LOS (*p* = 0.046), and fewer readmissions (*p* < 0.05). Intervention was safe and feasible.
Chen J et al. (2017) [30]	China	Assessor-blinded parallel-group RCT (1:1 allocation; intention-to-treat)	n = 54; ischemic/hemorrhagic; subacute	12-week program combining therapeutic exercise (1 h twice daily) + EMG-triggered neuromuscular stimulation (20 min twice daily). Telerehabilitation (videoconference supervision) vs. dose-matched face-to-face outpatient rehabilitation.	ADL/disability; balance; global disability; caregiver burden; muscle activation.	Primary: MBI. Secondary: BBS, mRS, CSI, EMG (RMS).	Significant within-group improvements in MBI, BBS, EMG activation, and CSI (time effect *p* < 0.001). No significant between-group differences (no group × time interaction; mRS *p* = 0.860 at 12 weeks, *p* = 0.278 at 24 weeks). No therapy-related adverse events.
Vloothuis JDM et al. (2019) [31]	Netherlands	Multicenter RCT (observer-blinded; intention-to-treat).	n = 66 patient–caregiver dyads; ischemic/hemorrhagic; subacute inpatient phase.	8-week caregiver-mediated task-specific mobility program (≥30 min/session, ≥5 days/week; ~150 min/week) supported by e-health/optional telecontact + usual care vs. usual care alone.	Primary: mobility; length of stay. Secondary: motor function, balance, ADL, psychosocial outcomes (patients and caregivers).	Primary: SIS 3.0 (mobility), LOS. Secondary: FMA-LE, BBS, BI, mRS, HADS, CSI.	No significant between-group effects for primary outcomes (SIS-mobility *p* = 0.229 at 8 weeks; *p* = 0.961 at 12 weeks; LOS *p* = 0.818). No differences in most functional secondary outcomes. Significant reductions in patient anxiety (*p* = 0.023; maintained at 12 weeks *p* = 0.009) and caregiver depression (*p* = 0.003). Intervention feasible and safe.
Chen J. et al. (2020) [33]	China	Randomized controlled trial	n = 52; first-ever subcortical stroke; subacute.	12-week program (10 sessions/week): 60 min OT/PT + 20 min EMG-triggered NMES per session. Home-based telerehabilitation with live videoconference supervision vs. dose-matched outpatient conventional rehabilitation.	Motor function; ADL; neuroplasticity (functional/structural MRI).	Primary: FMA (UE + LE), MBI. Secondary: rsFC (M1–M1), VBM, CST integrity (DTI).	Greater motor improvement in telerehabilitation vs. control (FMA *p* = 0.011). Increased interhemispheric connectivity (rsFC *p* = 0.031). MBI non-inferior (no superiority). Effects not maintained at 12-week follow-up. No adverse events.
Salgueiro C et al. (2020) [38]	Spain	Prospective pilot controlled trial. Non-randomized follow-up.	n = 49; ischemic/hemorrhagic; subacute	3-month home-based trunk/core stability exercises delivered via telerehabilitation app vs. usual care. No fixed dosage; voluntary use.	Balance (sitting and standing), gait, falls, feasibility and adherence	Primary: S-TIS 2.0, S-FIST. Secondary: BBS, S-PASS, 3-BBA, gait analysis.	Both groups improved over time. Greater improvements in balance and gait in the intervention group, but no statistically significant between-group differences. Low adherence (~31%). Feasible as adjunct to usual care.
Wu Z et al. (2020) [37]	China	Two-arm parallel RCT (assessor-blinded)	n = 64; ischemic/hemorrhagic; acute stroke	Post-discharge internet-based multidisciplinary telerehabilitation (videoconference, 2×/week) including progressive motor, balance, gait, and ADL training vs. weekly telephone follow-up.	Motor function, balance, gait/mobility & endurance, ADL independence, quality of life	FMA, BBS, TUG, 6MWT, MBI, SS-QOL.	Significant group × time effects favoring telerehabilitation across motor, balance, mobility, ADL, and QoL outcomes (all *p* < 0.001). Higher FMA, BBS, and SS-QOL scores at 12 weeks. Intervention safe and effective.
Bellomo RG et al. (2020) (WeReha) [36]	Italy	Quasi-experimental pre–post clinical study	n = 22; first-ever ischemic stroke; chronic phase.	12-week home-based biofeedback/exergaming program combining balance and upper/lower-limb motor training (≥3 sessions/week; ≥15–30 min/session) with remote monitoring; no concurrent conventional therapy.	ADL/functional independence; balance; motor impairment; global disability; technology acceptance.	BI, BBS, FM, mRS; TAM.	Significant pre–post improvements in BI (*p* = 0.036), BBS (*p* = 0.008), FMA (*p* = 0.003), and mRS (*p* = 0.047), with medium-to-large effect sizes. Gains largely maintained at follow-up (except slight BI decline). No adverse events. High technology acceptance.
Chen SC et al. (2021) [26]	Taiwan	Pilot RCT	n = 30; ischemic/hemorrhagic; chronic.	4-week program (3×/week, 40 min/session). Kinect-based balance telerehabilitation with remote monitoring vs. dose-matched conventional physiotherapy.	Balance, functional mobility, fear of falling, lower-limb strength, gait ability	BBS, TUG, MFES, Motricity Index (LE), FAC.	Both groups improved significantly in balance (BBS). Telerehabilitation group improved functional mobility (TUG). No significant between-group differences. Intervention feasible and safe.
Hernandez A et al. (2022) [46]	Canada	Evaluator-blinded, parallel RCT (before–after–follow-up)	n = 51; mixed stroke; chronic.	4-week home-based VR upper-limb training (≥20 min/session, 5×/week) with remote monitoring vs. self-directed GRASP home exercise program.	Upper-limb motor function; arm use/impact; feasibility/adherence.	Primary: FMA-UE. Secondary: SIS 3.0, MAL-14.	No significant between-group differences. Significant time effects for FMA-UE and SIS. Higher proportion reaching MCID in VR group (35% vs. 20%), particularly among high users. Gains not clearly maintained at follow-up. No serious adverse events.
Garcia A. et al. (2022) (Muvity) [35]	Spain	Feasibility randomized cross-over trial	n = 10 chronic post-stroke	8-week home-based non-immersive VR upper-limb exergaming (30 min/session, 3×/week) with remote monitoring vs. self-directed conventional home exercise program (dose-matched).	Feasibility/acceptability; ADL function; pain; balance; health status; kinematics (ROM).	FIM, VAS (pain), BBS, SF-36 (PCS), ROM measures; usability questionnaire.	No statistically significant between-group differences. Trends favored VR for ADL and pain (low–moderate effect sizes). No clear balance or QoL differences. High usability and motivation reported.
Alhusayni AI et al. (2023) [27]	Scotland (United Kingdom)	Randomized feasibility study (2-arm; assessor not blinded).	n = 26; ischemic/hemorrhagic; early subacute inpatient.	4-week individualized video-based upper-limb/trunk exercise program (≤30 min/session, 5×/week) delivered via online platform + usual care vs. usual inpatient physiotherapy alone.	Feasibility (recruitment, adherence, safety); upper-limb and trunk function; spasticity; acceptability.	ARAT; TIS; MAS; feasibility and acceptability metrics.	Intervention feasible and safe. Clinical outcomes improved in both groups. Trend toward greater upper-limb improvement with adherence (more participants exceeding ARAT MCID in intervention group). No significant spasticity changes. Adverse events not intervention-related.
Federico S. et al. (2023) [43]	Italy	Multi-center longitudinal pilot, pre–post	n = 74; first-ever ischemic stroke; post-hospital phase (2–18 months).	4-week synchronous home-based telerehabilitation (20 sessions/domain; 1 h/session, 5×/week) targeting motor, cognitive, and/or speech-language domains via VR platform.	Upper-limb motor function and dexterity, balance, ADL independence, cognition, language (if treated), HRQoL, depressive symptoms	FMA-UE, NHPT, BI, MoCA, AAT, SF-36, BDI.	Significant pre–post improvements in motor function, dexterity, balance, ADL independence, cognition, and physical HRQoL. No significant change in depressive symptoms. Greater gains observed in multi-domain interventions. No safety concerns reported.
Özden F et al. (2024) [34]	Turkey	Double-blind RCT	n = 30; ≥1-month post-stroke (mostly chronic).	6-week home-based balance/coordination program (twice daily) delivered via video-based telerehabilitation vs. paper-based prescription (dose-matched).	Balance confidence; fear of falling/falls efficacy; stroke self-efficacy/functional independence; satisfaction	FES-I; ABC; SSEQ; TSQ (intervention only).	Both groups improved (within-group *p* < 0.05). Greater improvements in video telerehabilitation group for balance confidence (ABC *p* = 0.042) and self-efficacy (SSEQ *p* = 0.018). No between-group difference in FES-I. High satisfaction reported.
Ali HM et al. (2024) [42]	Nigeria	Parallel 2-arm pilot study (blinded outcome assessors).	n = 14; ischemic/hemorrhagic; chronic.	4-week audio-based telerehabilitation (3–5 calls/week) combined with task-oriented home upper-limb training vs. usual outpatient care with home program only.	Upper-limb function; ADL; quality of life; feasibility/safety	FMA-UE; WMFT; WHOQOL-BREF; BI.	Significant between-group improvements favoring Telerehabilitation for motor outcomes (FMA-UE *p* = 0.02; WMFT *p* = 0.03). QoL improved only in environmental domain (*p* = 0.001). No significant BI change (*p* = 0.49). High adherence (100%) and no adverse events.
Saygili F et al. (2024) [45]	Turkey	Evaluator-blinded RCT (1:1 allocation)	n = 18; ischemic/hemorrhagic; ≥1-month post-stroke.	3-week Tele-CIMT (90 min/day, 5×/week; 22.5 h supervised) delivered via videoconference + home exercise program vs. home exercise program alone (dose-matched).	Upper-limb motor function; dexterity; strength; arm use; ADL independence.	Primary: STREAM, FM-UE, WMFT, 9-HPT. Secondary: grip/pinch strength, MAL-28, FIM.	Significant group × time interactions favoring Tele-CIMT across motor, dexterity, strength, arm use, and ADL outcomes (all *p* < 0.05). Clinically meaningful improvements exceeding MCID for STREAM, FM-UE, WMFT, and 9-HPT. No adverse events; high adherence.
Kintrilis N et al. (2024) [40]	Greece	Three-arm RCT.	n = 90; ischemic/hemorrhagic; acute inpatient stroke.	12-week resistance training program (30 min/session, 3×/week, moderate intensity) delivered face-to-face or via teleconferencing vs. usual care.	Quality of life, mobility, balance, lower-limb strength and endurance, cardiorespiratory efficiency	EQ-5D (VAS), TUG, BBS, CST, 6MWT, A–VO_2_.	Intervention groups showed significant improvements in quality of life (*p* = 0.009; telerehab *p* = 0.044) and cardiorespiratory efficiency (*p* = 0.007). No significant differences between face-to-face and telerehabilitation delivery. Functional improvements observed over time.
Wang Y et al. (2024) [44]	China	Controlled parallel-group trial	n = 80; ischemic/hemorrhagic; subacute	12-week internet-based remote home rehabilitation (30 min twice daily, 6×/week) with video supervision and wearable monitoring vs. structured home exercise program with follow-up.	Motor function; balance; ADL; gait parameters; mood; neurotrophic biomarkers.	FMA, MAS, BBS, MBI, HADS; gait analysis; serum BDNF, NT-3, NGF.	No baseline differences. At 4 and 12 weeks, significantly greater improvements in the intervention group across motor function, balance, ADL, gait, spasticity, mood, and neurotrophic markers (*p* < 0.05; mostly *p* < 0.001).
Lee HC et al. (2025) [41]	Taiwan	Pilot RCT	n = 24; ischemic/hemorrhagic; acute inpatient.	Inpatient telerehabilitation program (10 sessions) delivered remotely vs. dose-matched conventional inpatient rehabilitation.	ADL, mobility, quality of life, emotional status	MBI; PASS; FAC; PHQ-9.	No significant between-group differences. Both groups improved in ADL (MBI) and postural control (PASS). Telerehabilitation showed greater walking improvement (FAC), while control improved more in depressive symptoms (PHQ-9). Intervention feasible; findings preliminary.

Abbreviations: AAT, Aachener Aphasia Test; ABC, Activities-specific Balance Confidence Scale; ARAT, Action Research Arm Test; A–VO_2_, Arteriovenous Oxygen Difference; BBDNF, Brain-Derived Neurotrophic Factor; BBS, Berg Balance Scale; BDI, Beck Depression Inventory; BI, Barthel Index; CSI, Caregiver Strain Index; CST, Chair Stand Test; DTI, Diffusion Tensor Imaging; EQ-5D, EuroQol-5 Dimensions; FA, Fractional Anisotropy; FAC, Functional Ambulation Category; FES, Falls Efficacy Scale; FES-I, Falls Efficacy Scale–International; FIM, Functional Independence Measure; FM, Fugl-Meyer Assessment; FMA-LE, Fugl-Meyer Assessment for the Lower Extremity; FMA-UE, Fugl-Meyer Assessment for the Upper Extremity; GRASP, Graded Repetitive Arm Supplementary Program; HADS, Hospital Anxiety and Depression Scale; HEP, Home Exercise Program; LOS, Length of Stay; MAL, Motor Activity Log; MAL-28, Motor Activity Log–28 items; MAS, Modified Ashworth Scale; MBI, Modified Barthel Index; MD, Mean Diffusivity; MFES, Modified Falls Efficacy Scale; MI, Motricity Index; MoCA, Montreal Cognitive Assessment; mRS, Modified Rankin Scale; NGF, Nerve Growth Factor; NHPT, Nine-Hole Peg Test; NT-3, Neurotrophin-3; PASS, Postural Assessment Scale for Stroke; PHQ-9, Patient Health Questionnaire-9; RMS, Root Mean Square; rsFC, Resting-State Functional Connectivity; SF-36, 36-Item Short Form Health Survey; SIS, Stroke Impact Scale; SIS 3.0, Stroke Impact Scale version 3.0; SS-QOL, Stroke-Specific Quality of Life Scale; SSPSC, Stroke-Specific Patient Satisfaction with Care; STREAM, Stroke Rehabilitation Assessment of Movement; SSEQ, Stroke Self-Efficacy Questionnaire; S-TIS, Spanish Trunk Impairment Scale; TAM, Technology Acceptance Model; TIS, Trunk Impairment Scale; TSQ, Telemedicine Satisfaction Questionnaire; TUG, Timed Up and Go Test; VBM, Voxel-Based Morphometry; WMFT, Wolf Motor Function Test; 6MWT, 6-Minute Walk Test.

## Data Availability

No new data were created or analyzed in this study. Data sharing is not applicable to this article.

## Abbreviations

The following abbreviations are used in this manuscript:

ADL	Activities of Daily Living
BI	Barthel Index
BBS	Berg Balance Scale
EQ-5D	EuroQol-5 Dimensions
FAC	Functional Ambulation Category
FES	Falls Efficacy Scale
FES-I	Falls Efficacy Scale–International
FIM	Functional Independence Measure
FMA	Fugl-Meyer Assessment
FMA-UE	Fugl-Meyer Assessment for the Upper Extremity
FMA-LE	Fugl-Meyer Assessment for the Lower Extremity
GRADE	Grading of Recommendations Assessment, Development and Evaluation
HADS	Hospital Anxiety and Depression Scale
HEP	Home Exercise Program
MAS	Modified Ashworth Scale
MBI	Modified Barthel Index
MCID	Minimal Clinically Important Difference
PASS	Postural Assessment Scale for Stroke
PHQ-9	Patient Health Questionnaire-9
PRISMA	Preferred Reporting Items for Systematic Reviews and Meta-Analyses
RCT	Randomized Controlled Trial
SIS	Stroke Impact Scale
SIS 3.0	Stroke Impact Scale version 3.0
SS-QOL	Stroke-Specific Quality of Life Scale
SSPSC	Stroke-Specific Patient Satisfaction with Care
STREAM	Stroke Rehabilitation Assessment of Movement
TUG	Timed Up and Go Test
WMFT	Wolf Motor Function Test
10MWT	10-Meter Walk Test
6MWT	6-Minute Walk Test
